# Supplementary material for: Light-mediated discovery of surfaceome nanoscale organization and intercellular receptor interaction networks
Source: Nat Commun. 2021 Dec 2;12:7036. doi: 10.1038/s41467-021-27280-x (PMC8639842; doi:10.1038/s41467-021-27280-x)
Supplement: Supplementary file 1 — Supplementary Information [file 41467_2021_27280_MOESM1_ESM.pdf]

SUPPLEMENTARY INFORMATION

Light-mediated discovery of surfaceome nanoscale organization and intercellular receptor interaction networks

Maik Müller[1,2], Fabienne Gräbnitz[3], Niculò Barandun[3], Yang Shen[4], Fabian Wendt[1,2], Sebastian N. Steiner[1,2], Yannik Severin[5], Stefan U. Vetterli[6], Milon Mondal[6], James R. Prudent[7], Raphael Hofmann[8], Marc van Oostrum[1,2], Roman C. Sarott[8], Alexey I. Nesvizhskii[9,10], Erick M. Carreira[8], Jeffrey W. Bode[8], Berend Snijder[5,2], John A. Robinson[6], Martin J. Loessner[4], Annette Oxenius[3], Bernd Wollscheid[1,2*]

[1]Department of Health Sciences and Technology (D-HEST), ETH Zurich, Institute of Translational Medicine (ITM), Zurich, Switzerland
[2]Swiss Institute of Bioinformatics (SIB), Lausanne, Switzerland
[3]Department of Biology, ETH Zurich, Institute of Microbiology, Zurich, Switzerland
[4]Institute of Food Nutrition and Health, Department of Health Sciences and Technology, ETH Zurich, Zurich, Switzerland
[5]Institute of Molecular Systems Biology, Department of Biology, ETH Zurich, Zurich, Switzerland
[6]Chemistry Department, University of Zurich, Zurich, Switzerland
[7]Centrose LLC, Madison, Wisconsin, USA
[8]Laboratory of Organic Chemistry, Department of Chemistry and Applied Biosciences, ETH Zurich, Zurich, Switzerland
[9]Department of Pathology, University of Michigan, Ann Arbor, MI, USA
[10]Department of Computational Medicine and Bioinformatics, University of Michigan, Ann Arbor, MI, USA
*Correspondence should be addressed to B.W. (wbernd@ethz.ch)

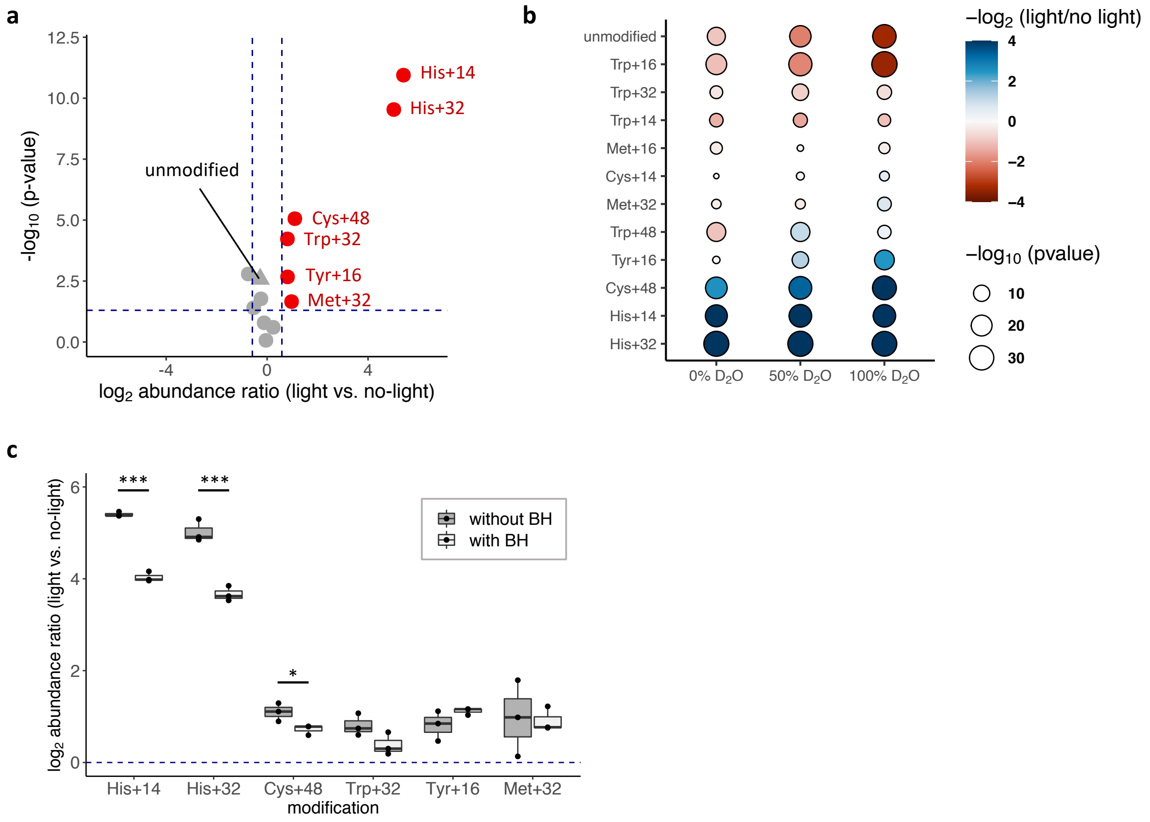

Supplementary Figure 1. **Identification and tuning of light-induced biotin-hydrazide reactive protein modifications. a**, Volcano plot showing relative abundance changes of transferrin amino acid modifications as quantified by mass spectrometry before and after illumination for 15 min (n=3 biologically independent samples), tested using a two-sided Student's t-test. Dots and triangles represent modified and unmodified transferrin proteins, respectively. Grey and red indicates non-regulated and significantly regulated proteins. **b**, D_2O enhanced protein modification. Dot plot showing relative conversion of modified or unmodified transferrin peptides after illumination for 30 min in PBS with increasing D_2O concentrations. **c**, Boxplot showing quantitative generation of modified transferrin proteins after illumination for 15 min in the absence or presence of biocytin-hydrazide (BH) linker (n=3 biologically independent samples). The center line of box-plots represents the median, box limits the first and third quartiles, whiskers delimit the last data points within 1.5-fold interquartile range and dots any outlier data points. Statistical analysis was done with two-tailed unpaired Student's t test. One star indicates $P < 0.1$, two stars $P < 0.01$ and three stars $P < 0.005$. Source data are provided as a Source Data file.

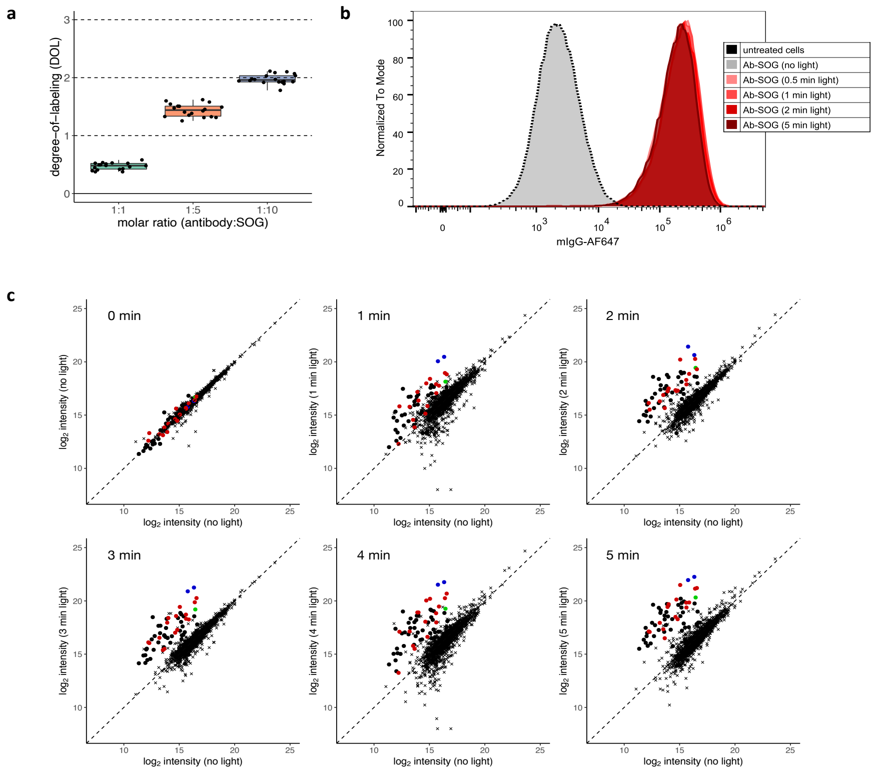

Supplementary Figure 2. Illumination-dependent fine-tuning of CD20 receptor microenvironment mapping using antibody-guided LUX-MS. a, Box plot showing number of thiorhodamine SOG molecules per IgG antibody at different molar coupling ratios. The center line of box-plots represents the median, box limits the first and third quartiles, whiskers delimit the last data points within 1.5-fold interquartile range and dots any outlier data points (n=18 technical replicates per ratio). **b**, Histogram plot showing binding of singlet oxygen generator coupled anti-CD20 antibodies to human B-lymphoma (SUDHL6) cells that were subsequently illuminated for 0 to 5 min (>50’000 cells per condition). **c**, Scatter plots showing relative abundance of LUX-MS quantified proteins from anti-CD20 Ab-SOG treated B-lymphoma SUDHL6 cells between a non-labeled control (x-axis) or samples illuminated for 0 - 5 min (y-axis). Dots and crosses represent cell surface and otherwise annotated proteins, respectively. Blue, green and red indicate chains of the anti-CD20 antibody, the primary binding target CD20 and known CD20 associated surface proteins. Source data are provided as a Source Data file.

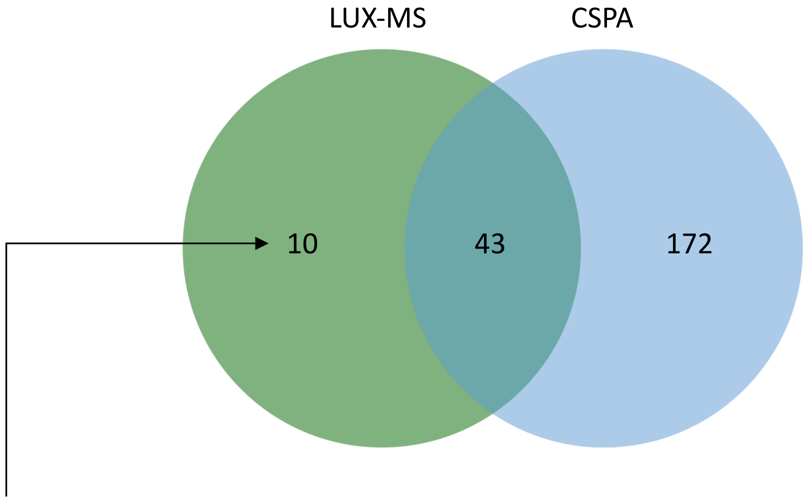

UniProt ID	Protein	Name	N-glycosylation
O14638	ENPP3 (CD203c)	Ectonucleotide pyrophosphatase/phosphodiesterase family member 3	yes
O94985	CLSTN1	Calsyntenin-1 (Alzheimer-related cadherin-like protein)	yes
P01780	IGHV3-7	Immunoglobulin heavy variable 3-7	
P01834	IGKC	Immunoglobulin kappa constant	
P01906	HLA-DQA2	HLA class II histocompatibility antigen, DQ alpha 2 chain	yes
P01871	IGHM	Immunoglobulin heavy constant mu	yes
P53985	SLC16A1	Monocarboxylate transporter 1 (MCT 1) (Solute carrier family 16 member 1)	
P60033	CD81	Tetraspanin-28	
Q01650	SLC7A5 (CD98 lc)	Large neutral amino acids transporter small subunit 1 (Solute carrier family 7 member 5)	
Q14392	LRRC32	Transforming growth factor beta activator LRRC32	yes

Supplementary Figure 3. **Surfaceome detection by LUX-MS is not restricted to protein N-glycosylation**. Venn diagram showing overlap of cell surface proteins identified by anti-CD20 antibody-guided LUX-MS and Cell Surface Capture as reported by the Cell Surface Protein Atlas (CSPA, https://wlab.ethz.ch/cspa/) on B-lymphoma SUDHL6 cells. Cell surface proteins specifically identified by LUX-MS are shown with additional information such as N-glycosylation state. Source data are provided as a Source Data file.

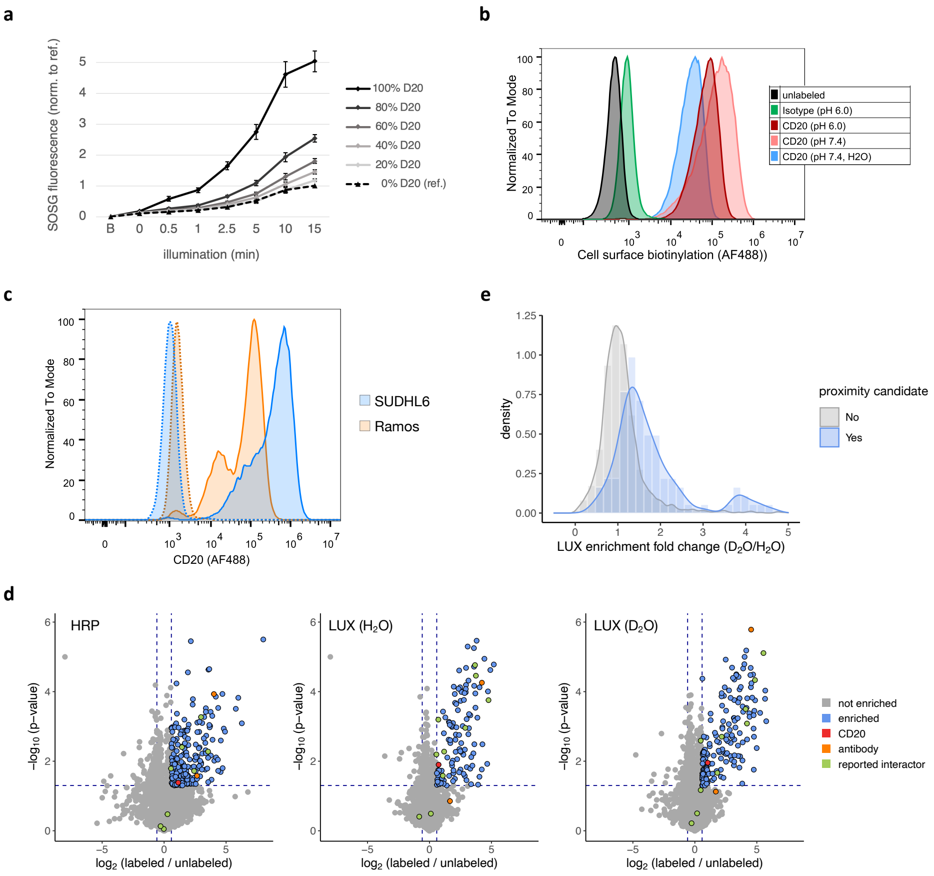

Supplementary Figure 4. Buffer and catalyst-dependent fine-tuning of CD20 receptor microenvironment mapping using antibody-guided LUX-MS. a, Profile plots showing light-dependent fluorescence of oxidized singlet oxygen sensor green (SOSG) at increasing D_2O/H_2O ratios as read-out for photo-oxidation efficiency. Data are presented as mean values +/- SD (n=3 biologically independent samples). **b**, Histogram plot showing cell surface biotinylation of B-lymphoma SUDHL6 cells treated with SOG-coupled Isotype control (Isotype) or anti-CD20 antibody (CD20) prior light-induced LUX-labeling in D_2O at pH 6 or 7.4 or H_2O at pH 7.4 (H_2O) (>50’000 cells per condition). **c**, Histogram plot showing CD20 surface expression on B-lymphoma SUDHL6 cells and resting B-cells (Ramos). **d**, Volcano plots showing relative quantitative enrichment of proteins proximity labeled by horseradish peroxidase (HRP) or thiorhodamine (SOG) coupled anti-CD20 antibodies), tested using a two-sided Student’s t-test. SOG-based LUX-labeling was thereby performed in either water (H_2O) and heavy water (D_2O) based buffer. **e**, Histogram plot showing the change in LUX-enrichment of identified CD20 proximity candidates or other proteins when switching from water (H_2O) to heavy water (D_2O) based buffer (>12’000 cells per condition). Source data are provided as a Source Data file.

a

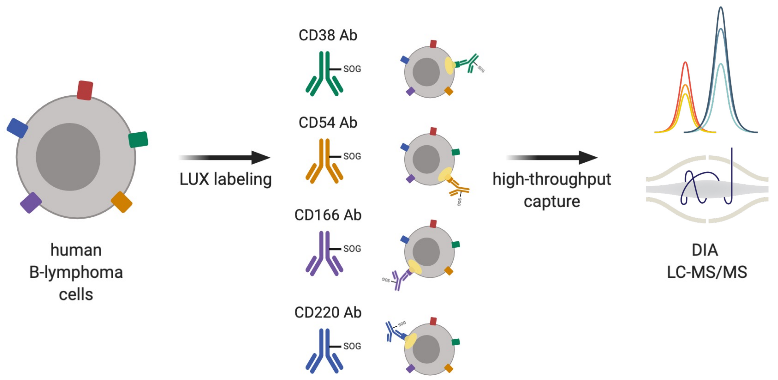

b

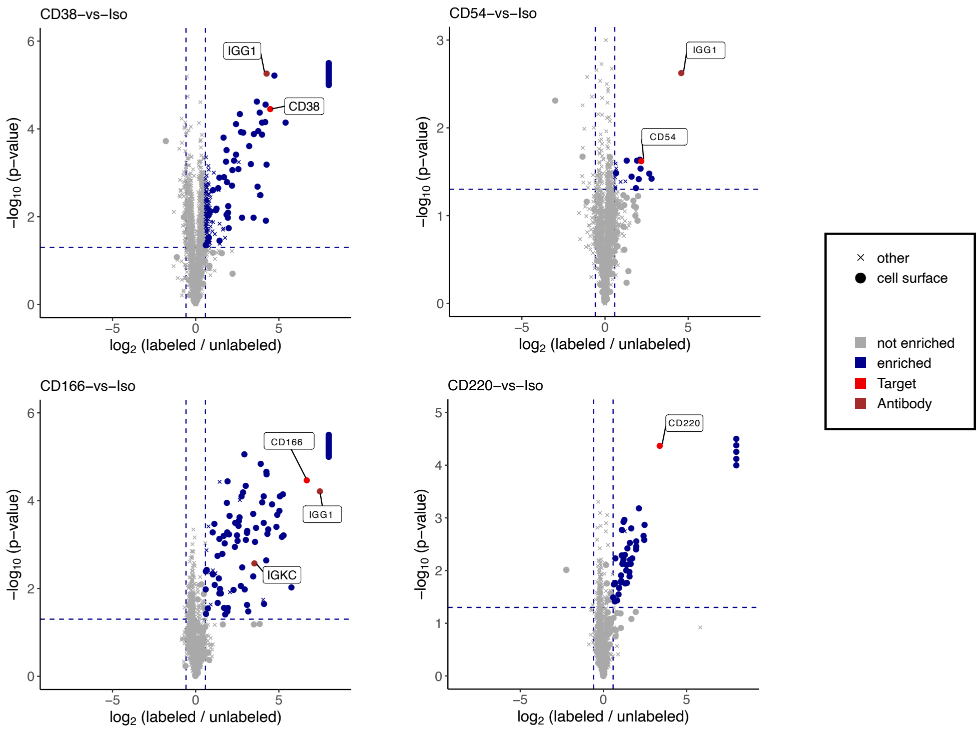

Supplementary Figure 5. **Elucidation of distinct surfaceome receptor neighborhoods on living cells. a**, Schematic outline of mapping distinct surfaceome receptor neighborhoods on living cells using a selection of singlet oxygen generator (SOG)-coupled antibodies and data-independent acquisition (DIA) mass spectrometry. **b**, Volcano plots showing relative abundance changes of LUX-MS quantified proteins from B-lymphoma SUDHL6 cells treated with either SOG-coupled isotype control (Iso), anti-CD38, anti-CD54, anti-CD166 or anti-CD220 antibodies and illuminated for 15 min, tested using a two-sided Student's t-test. Dots and crosses represent cell surface and otherwise annotated proteins, respectively. Brown, red and blue indicate chains of the used antibody, the primary binding target and cell surface proximal candidates. Source data are provided as a Source Data file.

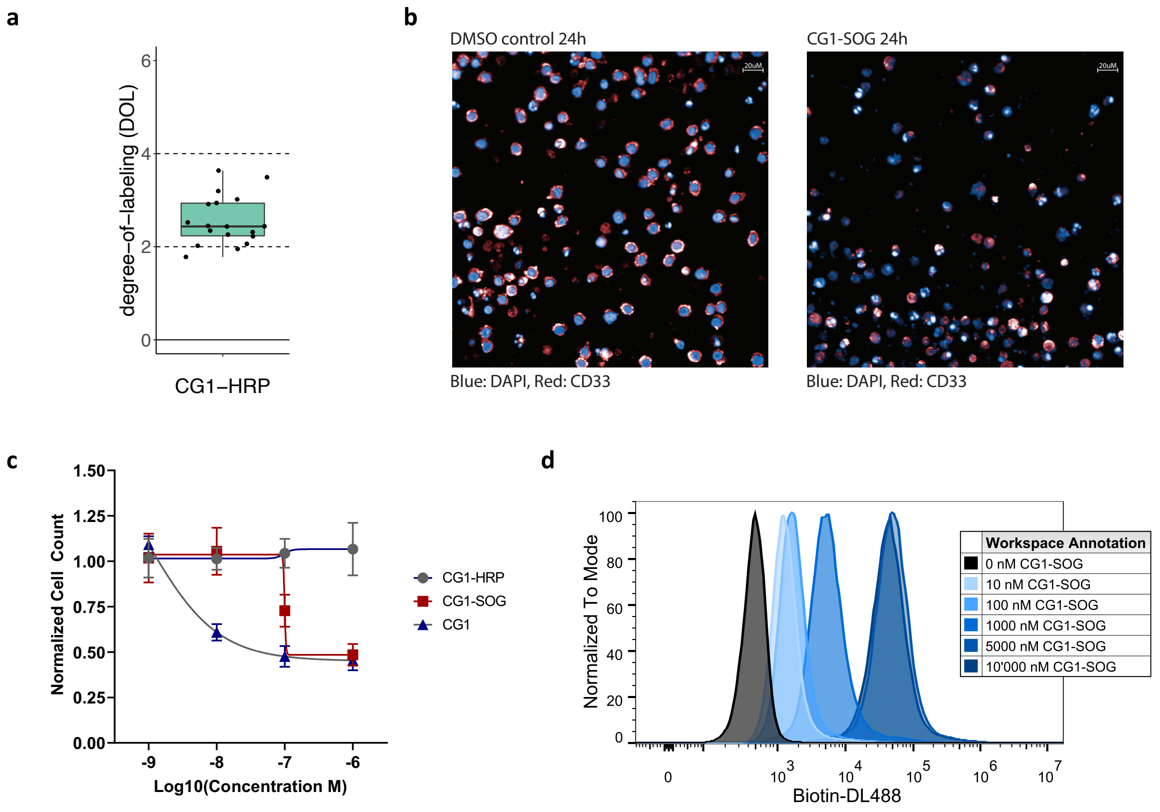

Supplementary Figure 6. Activity-preserving coupling of SOGs to small molecule drugs. **a**, Boxplot showing degree-of-labeling (DOL) of horseradish peroxide (HRP) enzymes coupled to cytotoxic small molecule drug CG1 at indicated molar ratios. The center line of box-plots represents the median, box limits the first and third quartiles, whiskers delimit the last data points within 1.5-fold interquartile range and dots any outlier data points (n=18 technical replicates). **b**, Representative immunofluorescence images of single cell chemosensitivity screen of human promyelocytic leukemia (HL60) cells treated with DMSO or SOG-coupled CG1 (CG1-SOG) for 24 hours and stained for nuclei (blue, DAPI) and myeloid marker protein CD33 (red) (n=10 technical replicates). **c**, Profile plot of single cell chemosensitivity screen showing normalized HL60 cell count after treatment with either CG1, HRP-coupled CG1 (CG1-HRP) or SOG-coupled CG1 (CG1-SOG) for 24 h at indicated concentrations. Data are presented as mean values +/- SD (n=10 technical replicates). **d,** Histogram plot showing light-induced cell surface biotinylation of HL60 cells treated with indicated concentrations of CG1-SOG after illumination for 15 min in the presence of biocytin-hydrazide linker (>45'000 cells per condition). Source data are provided as a Source Data file.

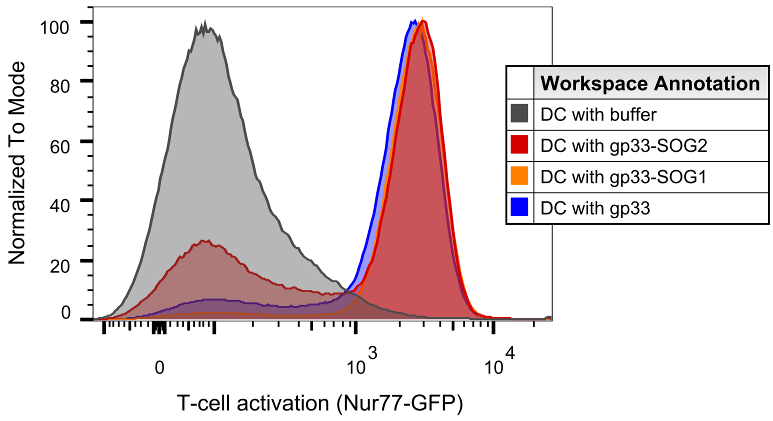

Supplementary Figure 7. Singlet oxygen generators (SOG) coupling does not affect immunogenicity of gp33. Histogram plot showing activation-indicating expression of Nur77-GFP of gp33-reactive $CD8^+$ T-cells after co-culture for 3 h with dendritic cells loaded with either buffer, underivatized gp33, gp33 with one SOG (gp33-SOG1) or gp33 with two SOG (gp33-SOG2) (>200'000 cells per condition).

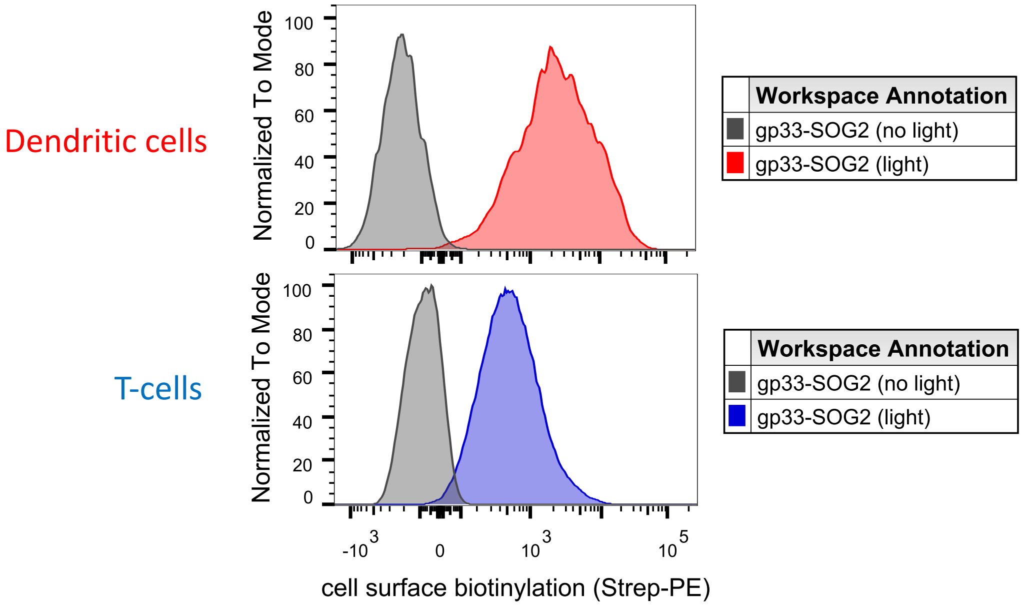

Supplementary Figure 8. **Light-activated proximity labeling of dynamic immune cell interactions**. Histogram plots showing cell surface biotinylation of gp33-SOG2 presenting dendritic cells and $CD8^+$ T-cells that were co-cultured for 3 h and LUX-labeled with or without illumination for 15 min (>5’000 cells per condition).

a

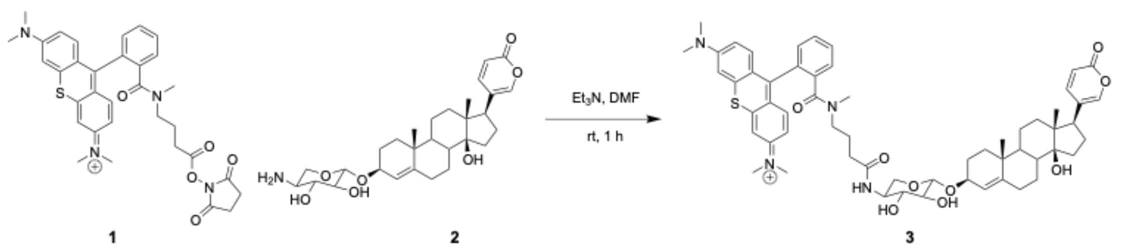

b

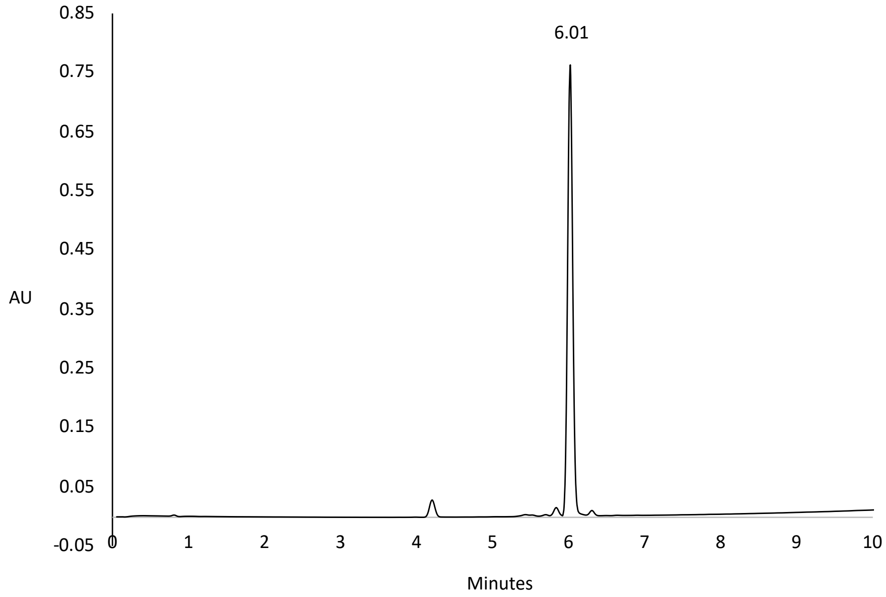

Supplementary Figure 9. **Synthesis and characterization of CG1-SOG. a,** Synthesis reaction of CG1-SOG. **b,** Analytical RP-HPLC of purified CG1-SOG. Analytical RP-HPLC was performed on a Acquity UPLC C18 column (1.7 µm, 2.1 mm I.D. x 150 mm) at 40 °C at a flow rate of 0.3 mL/min and a gradient of 5 - 95 % solvent B (ACN/H_2O + 0.1 % TFA) for 10 min.

A

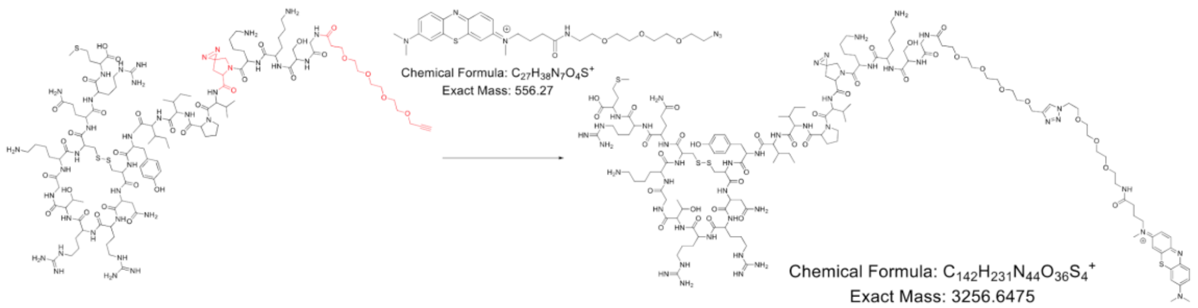

b

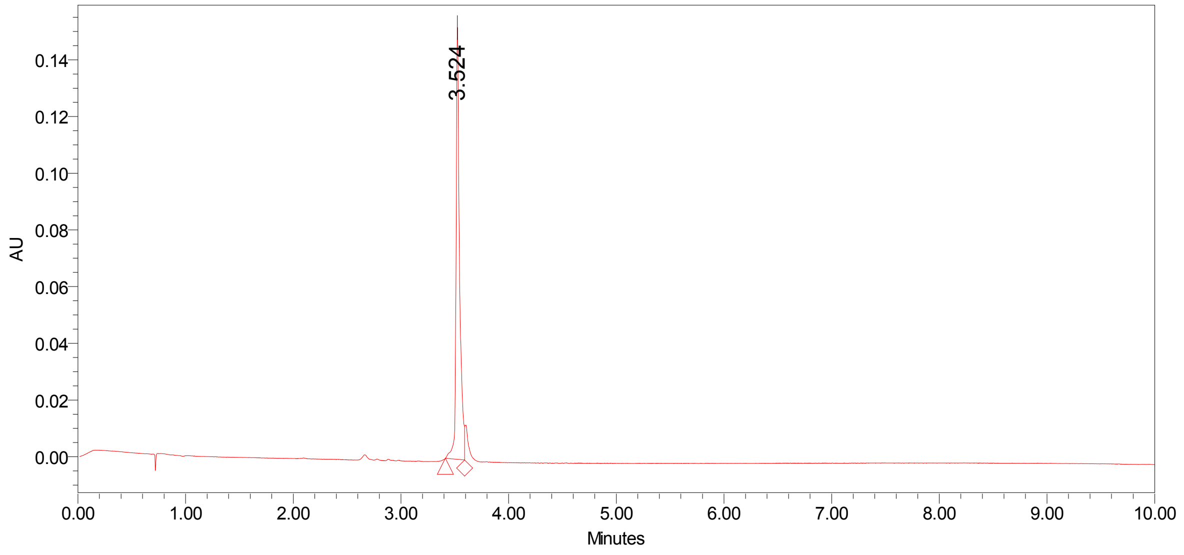

Supplementary Figure 10. **Synthesis and characterization of Thanatin-SOG. a,** Synthesis reaction of Thanatin-SOG. **b,** Analytical RP-HPLC of purified Thanatin-SOG. Analytical RP-HPLC was performed on a Acquity UPLC C18 column at 40 °C and a gradient of 5 - 95 % solvent B (ACN/H_2O + 0.1 % TFA) for 10 min.

a

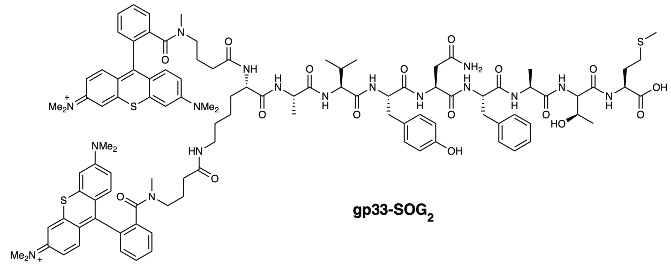

b

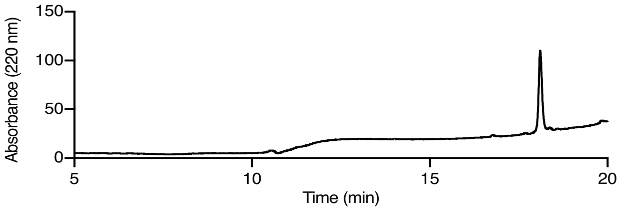

c

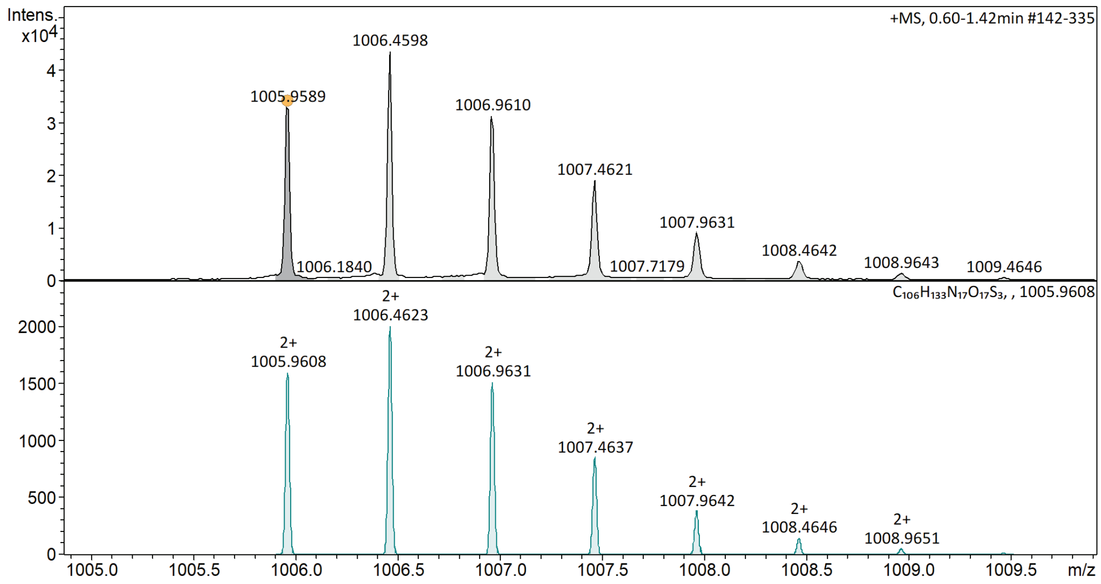

Supplementary Figure 11. **Characterization of gp33-SOG$_2$. a**, Chemical structure of gp33-SOG2. **b,** Analytical RP-HPLC of purified gp33-SOG$_2$. Analytical RP-HPLC was performed on a Shiseido C18 column (5 µm, 4.6 mm I.D. x 250 mm) at RT at a flow rate of 1 mL/min and a gradient of 10% solvent B for 3 min, 10 to 95% solvent B over 14 min, and 95% solvent B for 3 min. **c**, Observed (upper panel) and calculated (lower panel) HR-MS spectra. Spectra were obtained by the mass spectrometry service of the ETH Laboratory of Organic Chemistry on a Bruker Daltonics maXis ESI-QTOF spectrometer.

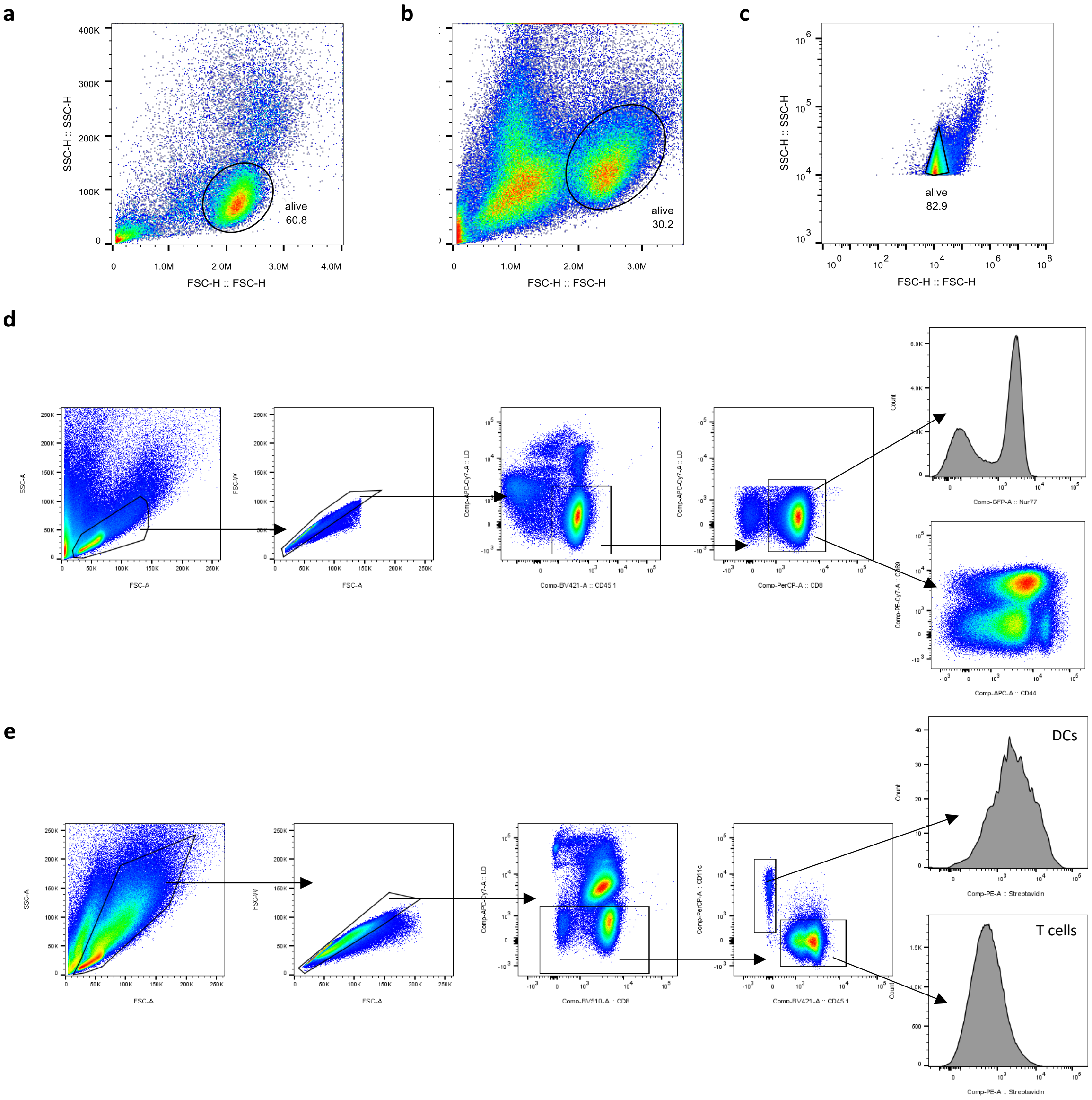

Supplementary Figure 12. **Gating strategies for flow cytometric analysis. a**, Sorting of human B-lymphoma (SUDHL6) cells surface biotinylated by anti-CD20 antibody-guided LUX-MS. **b**, Sorting of human promyelocytic leukemia (HL60) cells surface biotinylated by small molecule drug CG1-guided LUX-MS. **c**, Sorting of gram-positive *Listeria monocytogenes* bacteria cell surface biotinylated by intact virus bacteriophage-guided LUX-MS. **d**, Sorting of mouse primary CD8+ T-cells activated to express Nur77-GFP after incubation with antigen-loaded mouse dendritic cells. **e,** Sorting of mouse primary CD8+ T-cells and mouse dendritic cells from co-culture system after cell surface biotinylation by antigen gp33-guided LUX-MS.
