# Supplementary material for: Light-mediated discovery of surfaceome nanoscale organization and intercellular receptor interaction networks
Source: Nat Commun. 2021 Dec 2;12:7036. doi: 10.1038/s41467-021-27280-x (PMC8639842; doi:10.1038/s41467-021-27280-x)
Supplement: Supplementary file 3 — Description of Additional Supplementary Files [file 41467_2021_27280_MOESM3_ESM.docx]

File Name: Supplementary Data 1

Description: Interactive volcano plot showing relative abundance changes of LUX-MS quantified proteins from antiCD20-SOG treated B-lymphoma SUDHL6 cells with and without illumination for 5 min, tested using a two-sided Student’s t-test.

File Name: Supplementary Data 2

Description: Interactive volcano plot showing relative abundance changes of LUX-MS quantified proteins from CG1-SOG treated promyelocytic leukemia HL60 cells with and without illumination for 5 min, tested using a two-sided Student’s t-test.

File Name: Supplementary Data 3

Description: Interactive volcano plot showing relative abundance changes of LUX-MS quantified proteins from insulin-SOG and transferrin-SOG treated B-lymphoma SUDHL6 cells illuminated for 5 min, tested using a two-sided Student’s t-test.

File Name: Supplementary Data 4

Description: Interactive volcano plot showing relative abundance changes of LUX-MS quantified proteins from Thanatin-SOG treated Escherichia coli illuminated for 15 min with and without Thanatin competition, tested using a two-sided Student’s t-test.

File Name: Supplementary Data 5

Description: Interactive volcano plot showing relative abundance changes of LUX-MS quantified proteins from bacteriophage-SOG treated *Listeria monocytogenes* with and without illumination for 15 min, tested using a two-sided Student’s t-test.

File Name: Supplementary Data 6

Description: Interactive volcano plot showing relative abundance changes of LUX-MS quantified dendritic cell proteins from the two-cell systems with and without illumination for 15 min, tested using a two-sided Student’s t-test.

File Name: Supplementary Data 7

Description: Interactive volcano plot showing relative abundance changes of LUX-MS quantified T cell proteins from the two-cell systems with and without illumination for 15 min, tested using a two-sided Student’s t-test.
