# Supplementary material for: Light-mediated discovery of surfaceome nanoscale organization and intercellular receptor interaction networks
Source: Nat Commun. 2021 Dec 2;12:7036. doi: 10.1038/s41467-021-27280-x (PMC8639842; doi:10.1038/s41467-021-27280-x)
Supplement: Supplementary file 11 — Reporting Summary [file 41467_2021_27280_MOESM11_ESM.pdf]

nature research

Corresponding author(s): Bernd Wollscheid

Last updated by author(s): Jun 26, 2021

Reporting Summary

Nature Research wishes to improve the reproducibility of the work that we publish. This form provides structure for consistency and transparency in reporting. For further information on Nature Research policies, see our Editorial Policies and the Editorial Policy Checklist.

Statistics

For all statistical analyses, confirm that the following items are present in the figure legend, table legend, main text, or Methods section.

n/a	Confirmed	
☐	☒	The exact sample size (*n*) for each experimental group/condition, given as a discrete number and unit of measurement
☐	☒	A statement on whether measurements were taken from distinct samples or whether the same sample was measured repeatedly
☐	☒	The statistical test(s) used AND whether they are one- or two-sided<br>*Only common tests should be described solely by name; describe more complex techniques in the Methods section.*
☒	☐	A description of all covariates tested
☐	☒	A description of any assumptions or corrections, such as tests of normality and adjustment for multiple comparisons
☐	☒	A full description of the statistical parameters including central tendency (e.g. means) or other basic estimates (e.g. regression coefficient) AND variation (e.g. standard deviation) or associated estimates of uncertainty (e.g. confidence intervals)
☐	☒	For null hypothesis testing, the test statistic (e.g. *F*, *t*, *r*) with confidence intervals, effect sizes, degrees of freedom and *P* value noted<br>*Give P values as exact values whenever suitable.*
☒	☐	For Bayesian analysis, information on the choice of priors and Markov chain Monte Carlo settings
☒	☐	For hierarchical and complex designs, identification of the appropriate level for tests and full reporting of outcomes
☒	☐	Estimates of effect sizes (e.g. Cohen's *d*, Pearson's *r*), indicating how they were calculated

Our web collection on statistics for biologists contains articles on many of the points above.

Software and code

Policy information about availability of computer code

Data collection	Mass spectra were acquired using a Thermo Fisher Scientific mass spectrometers operated with the vendor provided Tune and Xcalibur software. Flow cytometry was performed on a Canto (BD Biosciences) and Accuri C6 Flow Cytometer (BD Biosciences) using vendor provided software. Microscopy images of the single cell chemosensitivity screen were acquired on a Opera Phenix automated spinning-disk confocal microscope sing vendor provided software.
Data analysis	Acquired spectra containing raw files were processed with either Trans Proteomic Pipeline (v4.7) using the Comet search engine (v.2015.01) or Proteome Discoverer (v.2.4 and v.2.5.0.400) using SEQUEST search engine (v.2.0.0.24) or FragPipe (v.9.4) using Crystal-C and MSfragger (v.20190628). Spectronaut (v.13) or Progenesis QI (v.4.0) was used for quantitative analysis of DIA and DDA identified peptides. Bioinformatic analysis was performed in the R statistical computing environment (v.3.4.0) using the R package MSstats (v.3.8.6) and results visualized using the packages ggplot2 (v.3.3.2) and plotly (v.4.9.2). Protein interaction networks were generated using Cytoscape (v.3.7.1) and protein structures visualized using PyMOL (v.2.4.0). Flow cytometry data was analyzed using FlowJo software (v.10.07). CellProfiler (v.2) was used for analysis of microscopy images of the single cell chemosensitivity screen.

For manuscripts utilizing custom algorithms or software that are central to the research but not yet described in published literature, software must be made available to editors and reviewers. We strongly encourage code deposition in a community repository (e.g. GitHub). See the Nature Research guidelines for submitting code & software for further information.

Data

Policy information about availability of data

All manuscripts must include a data availability statement. This statement should provide the following information, where applicable:

- Accession codes, unique identifiers, or web links for publicly available datasets
- A list of figures that have associated raw data
- A description of any restrictions on data availability

The mass spectrometry data generated in this study have been deposited to the ProteomeXchange Consortium (http://proteomecentral.proteomexchange.org) via the PRIDE partner repository under the accession code PXD020481 (http://www.ebi.ac.uk/pride/archive/projects/PXD020481). SwissProt-reviewed human protein databases were downloaded from (https://www.uniprot.org/). Source data are provided with this paper.

Field-specific reporting

Please select the one below that is the best fit for your research. If you are not sure, read the appropriate sections before making your selection.

[x] Life sciences [ ] Behavioural & social sciences [ ] Ecological, evolutionary & environmental sciences

For a reference copy of the document with all sections, see nature.com/documents/nr-reporting-summary-flat.pdf

Life sciences study design

All studies must disclose on these points even when the disclosure is negative.

Sample size	For experiments including mass spectrometry, sample size was not predetermined by statistical methods for relative quantitative mass spectrometry experiments. However protein quantities were modeled based on the intensity of at least two peptides using Tukey's median polish method ensuring robust label-free quantification that allows for statistical testing with sample size at least n=3 that is commonly employed for quantitative proteomics investigations. For the single cell chemosensitivity screen, 10 technical replicates were analysed per time point based on the previously established pharmacoscopy workflow.
Data exclusions	Peptide identifications mapping to decoy or contaminant proteins or internal reference peptides were excluded from further analysis. Additionally, peptides were filtered based on MS/MS identification score to ensure a false discovery rate of < 1%. Outliers based on principal component analysis were removed to retain minimally two biological replicates per condition.
Replication	Overall, the anti-CD20 antibody-guided LUX-MS experiments were performed three times with increased replication and demonstrated the reproducibility of the optoproteomic workflow. Labeling and identification of extracellular protein interactions by LUX-MS was validated using bioorthogonal methods such as flow cytometry and confocal microscopy, and literature-based knowledge mining, respectively. Furthermore, small molecule, biomolecule, pathogen and immunogen guided LUX-MS was performed in at least two independent experiments with high overlap of identified proximity candidates. Cell surface localization of phage-LUX identified Listeria monocytogenes proteins was verified in two independent experiments using lysozyme-treated and untreated bacteria. Single cell chemosensitivitiy screen was performed with 10 technical replicates per time point (24 and 48 h of treatment).
Randomization	This study consists of multiple independent (LUX-MS) experiments with a smaller number of sample (n < 10) that were sequentially acquired by LC-MS/MS thereby minimizing technical variance and obviating the need for randomization.
Blinding	Investigators were not blinded to allocation of biological samples as the results of the mass spectrometry experiments are of technical nature and not prone to a potential observer bias.

Reporting for specific materials, systems and methods

We require information from authors about some types of materials, experimental systems and methods used in many studies. Here, indicate whether each material, system or method listed is relevant to your study. If you are not sure if a list item applies to your research, read the appropriate section before selecting a response.

Materials & experimental systems

n/a	Involved in the study	
[ ]	[x]	Antibodies
[ ]	[x]	Eukaryotic cell lines
[x]	[ ]	Palaeontology and archaeology
[ ]	[x]	Animals and other organisms
[x]	[ ]	Human research participants
[x]	[ ]	Clinical data
[x]	[ ]	Dual use research of concern

Methods

n/a	Involved in the study	
[x]	[ ]	ChIP-seq
[ ]	[x]	Flow cytometry
[x]	[ ]	MRI-based neuroimaging

Antibodies

Antibodies used	The following antibodies were used for flow cytometry analysis of murine cells: CD8a-BV510 (BioLegend, cat: 100752, clone: 53-6.7), CD45.1-PacificBlue (BioLegend, cat: 110722, clone: A20), CD44-APC (BD Biosciences, cat: 559250, clone: IM7), CD11c-PerCP (BioLegend, cat: 117326, clone: N418), CD69-PeCy7 (BioLegend, cat: 104512, clone: H1.2F3), CD25-FITC (BD Biosciences, cat: 553072, clone: 3C7).<br>The following antibodies were used for LUX-MS experiments: mouse IgG Isotype control antibody (Invitrogen, cat: 10400C), anti-human CD20 mouse IgG2 monoclonal antibody (Invitrogen, cat: 14-0209-82, clone: 2H7), anti-human CD38 chimeric monoclonal antibody kindly provided by Centrose LLC, anti-human CD54 mouse IgG1 monoclonal antibody (BioLegend, cat: 322704, clone: HCD54), anti-human CD166 mouse IgG1 monoclonal antibody (BioLegend, cat: 343902, clone: 3A6), anti-human CD220 mouse IgG2 monoclonal antibody (BioLegend, cat: 352602, clone: B6.220).<br>The following antibodies were used for single cell chemosensitivity screening: Alexa Fluor 647 anti-human CD33 mouse IgG1 antibody (BioLegend, cat: 366626, clone P67.6, lot: B306895).<br>The following antibodies were used for immunofluorescence microscopy: Alexa Fluor 555 anti-HA tag monoclonal antibody (Invitrogen, cat: 26183, clone: 2-2.2.14, lot: UC283116).
Validation	All commercial antibodies (except Alexa Fluor 555 anti-HA tag monoclonal antibody) were quality tested by flow cytometric analysis by the manufacturer. Alexa Fluor 555 anti-HA tag monoclonal antibody was quality tested by western blot and immunocytochemistry analysis by the manufacturer. The in-house developed anti-CD38 antibody was independently validated by Centrose LLC.

Eukaryotic cell lines

Policy information about cell lines

Cell line source(s)	Patient-derived B-lymphoma cell line SUDHL-6 (ATCC, CRL-2959), patient-derived promyelocytic leukemia cell line HL60 (ATCC CCL-240) and human Burkitt lymphoma B cell-line Ramos (ATCC, CRL-1596) were originally purchased from ATCC. Mouse dendritic cell line MutuDC1940 was established and provided by Hans Acha-Orbea (Department of Biochemistry, University of Lausanne, Switzerland; Fuertes Maracco et al. 2012, Front. Immunol.).
Authentication	Cell surface protein interactions on previously established and commonly used cell lines were investigated using LUX-MS and revealed cell type specific surface markers. No further authentication was applied.
Mycoplasma contamination	In the context of this study, cell lines were not specifically tested for Mycoplasma contamination.
Commonly misidentified lines<br>(See ICLAC register)	No commonly misidentified cell lines were used in this study.

Animals and other organisms

Policy information about studies involving animals; ARRIVE guidelines recommended for reporting animal research

Laboratory animals	P14 Nur77-GFP mice: Species: Mouse (mus musculus), name of line: B6.(SJL-Ptprca;B6/D2;C57BL/6)-Tg(TcrLCMV)327Sdz)-Tg(Nr4a1-EGFP/cre)820Khog/j, short name: P14 Nur77-GFP Ly5.1, sex: female and male, age: 6 - 16 weeks, dark/light cycle: 12h light/12h dark, ambient temperature: 22°C +/- 2°C, humidity: 55% +/- 10%
Wild animals	No wild animals were used in the study.
Field-collected samples	No field collected samples were used in the study.
Ethics oversight	All animal experiments were performed in accordance with institutional policies and Swiss federal regulations, following guidelines and being approved by the veterinary office of the Canton of Zürich (animal experimental permissions: 115/2017).

Note that full information on the approval of the study protocol must also be provided in the manuscript.

Flow Cytometry

Plots

Confirm that:

- [x] The axis labels state the marker and fluorochrome used (e.g. CD4-FITC).
- [x] The axis scales are clearly visible. Include numbers along axes only for bottom left plot of group (a 'group' is an analysis of identical markers).
- [x] All plots are contour plots with outliers or pseudocolor plots.
- [x] A numerical value for number of cells or percentage (with statistics) is provided.

Methodology

Sample preparation	FACS experiments in this study used human, murine and bacterial cells. Sample preparation is described in detail in the following methods sections: Antibody-guided surfaceome nanoscale mapping, Synthesis of singlet oxygen generator-coupled cardiac glycoside CG1, Identification of small molecule drug-targeted surfaceome structures, Bacteriophage-guided

	exploration of virus-targeted host surfaceomes and Flow cytometry analysis of murine immune cells.
Instrument	Flow cytometry was performed on a Canto (BD Biosciences) and Accuri C6 Flow Cytometer (BD Biosciences).
Software	FACS data was acquired using vendor provided software and analysed using FlowJo software (Treestar, Ashland, OR, USA).
Cell population abundance	Flow cytometry was used for analyzing light-induced cell surface biotinylation of LUX-labeled samples and did not yield in sorted cell populations.
Gating strategy	The gating strategy is outlined in the following methods sections: Antibody-guided surfaceome nanoscale mapping, Synthesis of singlet oxygen generator-coupled cardiac glycoside CG1, Identification of small molecule drug-targeted surfaceome structures, Bacteriophage-guided exploration of virus-targeted host surfaceomes and Flow cytometry analysis of murine immune cells.

☒ Tick this box to confirm that a figure exemplifying the gating strategy is provided in the Supplementary Information.
